# Morphological and Molecular Identification of Three New Macrofungal Species from Shenyang and Adjacent Areas, Northeast China

**DOI:** 10.3390/jof12070491

**Published:** 2026-07-03

**Authors:** Zi-Qi You, Lin-Jiang Zhou, Hai-Sheng Yuan, Hyang Burm Lee

**Affiliations:** 1 CAS Key Laboratory of Forest Ecology and Silviculture, Institute of Applied Ecology, Chinese Academy of Sciences, Shenyang 110164, China; youziqi0113@163.com (Z.-Q.Y.); zhoulj24@163.com (L.-J.Z.); 2 University of the Chinese Academy of Sciences, Beijing 100049, China; 3 Environmental Microbiology Lab, Department of Agricultural Biological Chemistry, College of Agriculture and Life Sciences, Chonnam National University, Gwangju 61186, Republic of Korea

**Keywords:** *Descolea*, *Leucocoprinus*, phylogeny, taxonomy, *Tephrocybe*

## Abstract

Macrofungal resources are abundant in Northeast China, but those from Shenyang and its surrounding areas remain insufficiently investigated. In this study, morphological and phylogenetic analyses were carried out on specimens collected from the vicinity of Shenyang, Liaoning Province. Phylogenetic trees were inferred using maximum likelihood and Bayesian inference methods based on sequence data from the nuclear ribosomal internal transcribed spacer (ITS) region and the nuclear ribosomal large subunit (LSU). Three new species, *Descolea laevis* (Bolbitiaceae), *Leucocoprinus shenyangensis* (Agaricaceae), and *Tephrocybe umbonata* (Lyophyllaceae), are described herein. *Descolea laevis* is characterized by pale yellow to light yellow basidiomata, a nearly smooth pileus surface, clavate to narrowly clavate cheilocystidia, fusiform to clavate pleurocystidia, and amygdaliform to limoniform, verrucose basidiospores. *Leucocoprinus shenyangensis* is distinguished by white to yellowish-white basidiomata, a pileus covered with light grey squamules, narrowly clavate to subcylindrical cheilocystidia, a hymenidermal pileipellis, and amygdaliform to limoniform basidiospores. *Tephrocybe umbonata* is characterized by orange-white to greyish-orange basidiomata, a pileus with a blunt umbo, a smooth or slightly finely fibrillose pileus surface, lageniform pleurocystidia with slightly acute apices, and amygdaliform to limoniform basidiospores. Detailed morphological descriptions, illustrations of microscopic structures, and phylogenetic evidence for the three new species are provided. The diagnostic characteristics separating the new taxa from their closely related species are also discussed.

## 1. Introduction

*Descolea* Singer is a small genus in Bolbitiaceae, Agaricales, and is typified by *D. antarctica* Singer [1]. Species of the genus are typically characterized by epigeous agaricoid basidiomata, a dry to viscid pileus surface, a central stipe, and ornamented basidiospores. *Descolea* is phylogenetically closely related to *Setchelliogaster*, *Descomyces*, and *Timgrovea*. However, *Setchelliogaster* forms secotioid basidiomata, whereas the latter two genera produce fully gasteroid basidiomata [2]. *Descolea* is generally regarded as an ectomycorrhizal genus associated with woody plants in forest ecosystems [2]. Species of the genus have been reported mainly from Australasia and southern South America, where they are often associated with forests dominated by Nothofagaceae and Myrtaceae [2]. However, Asian records suggest that the host range of the genus may be broader, and *D. quercina* has been reported in association with *Quercus* in moist temperate forests of Pakistan [3]. Approximately 29 species of *Descolea* are accepted worldwide according to Index Fungorum and MycoBank. To date, records of *Descolea* from China remain scarce, and only two species, *D. flavoannulata* (Lj. N. Vassiljeva) E. Horak and *D. pretiosa* E. Horak, have been confirmed [4].

*Leucocoprinus* Pat., typified by *L. cepistipes* (Sowerby) Pat., is a lepiotaceous genus in Agaricaceae [5]. Traditionally, *Leucocoprinus* was separated from *Leucoagaricus* by a combination of characters, including striate to plicate pileus margins, metachromatic basidiospores, pseudoparaphyses among the basidia, and the general absence of clamp connections [6,7]. Molecular phylogenetic studies have shown, however, that these characters are not fully congruent with natural lineages, and that *Leucocoprinus*, *Leucoagaricus*, *Micropsalliota*, and related genera form a complex assemblage within Agaricaceae [8]. Recent treatments have differed in their interpretation of this complex, ranging from a broad circumscription of *Leucocoprinus* that transfers many taxa formerly placed in *Leucoagaricus* to *Leucocoprinus* [9], to classifications that retain *Leucocoprinus* and *Leucoagaricus* as separate genera and recognize segregate genera such as *Candelolepiota* and *Macropsalliota* [10]. More recent treatments have further recognized additional segregate genera, including *Pulchrolepiota* and *Tristolepiota* [11]. These alternative classifications indicate that the generic limits of *Leucocoprinus* remain unsettled and that traditional morphological features should be used cautiously for generic delimitation. Ecologically, species assigned to *Leucocoprinus* and allied genera are saprotrophic and occur on diverse organic substrates, including forest soil, litter, humus, rotten wood, compost, flowerpots, greenhouses, and other decomposing plant materials [6,12]. The group is most diverse in tropical and subtropical regions [13], but recent studies from temperate areas of China suggest that its diversity in northern regions remains incompletely documented [14,15].

The circumscription of *Tephrocybe* has undergone substantial revision in recent years. Molecular phylogenetic studies have shown that *Tephrocybe* in the traditional broad sense is paraphyletic, and several species formerly assigned to this genus have been transferred to *Lyophyllum*, *Myochromella*, *Sagaranella*, *Sphagnurus*, and other allied genera [16]. At present, *Tephrocybe* Donk is treated as a genus in Lyophyllaceae, and it is typified by *T. rancida* (Fr.) Donk [17]. The presence of siderophilous granules in the basidia, a diagnostic character of Lyophyllaceae, is also consistently observed in *Tephrocybe* [18]. Molecular phylogenetic studies have shown that the core lineage of *Tephrocybe* is placed in the termitomycetoid clade, and it is closely related to *Termitomyces* and *Blastosporella* [19]. In contrast to these allied groups, *Tephrocybe* has clamp connections, and it is not associated with termites [20]. Subsequent studies have further shown that some taxa superficially resembling *Tephrocybe* should be segregated into newly established genera such as *Phaeotephrocybe* and *Praearthromyces*, indicating that a tephrocyboid habit alone is insufficient to define the genus. Ecologically, species of *Tephrocybe* are mainly free-living terrestrial fungi, and the type species *T. rancida* occurs on soil in coniferous forests [19]. Approximately 62 species of *Tephrocybe* are currently accepted worldwide, and the known diversity of the genus is concentrated mainly in temperate regions of the Northern Hemisphere, especially in Europe, whereas Asia and North America also contain several records, and the Southern Hemisphere is clearly less represented [21]. Nine species have been recorded from China, mainly from northern or temperate forest habitats.

Taken together, the contrasting trophic modes and substrate preferences of these genera suggest that heterogeneous forest environments may provide multiple ecological niches for macrofungi and harbor overlooked taxonomic diversity. Northeastern China contains extensive temperate forest ecosystems with diverse dominant tree species, litter inputs, and forest-floor microhabitats. However, macrofungal diversity in Shenyang and adjacent areas remains insufficiently investigated compared with that of several other regions of China. Field surveys were therefore conducted to document poorly known macrofungal diversity in local forest ecosystems, especially agaric taxa occurring on the forest floor in different habitat contexts. During these surveys, collections referable to the three genera mentioned above were obtained and confirmed to represent three previously undescribed species based on morphological characteristics and phylogenetic analyses of ITS and LSU sequences. The aim of the present study is to clarify the taxonomic identities and phylogenetic positions of these new taxa and to provide detailed descriptions, illustrations, and ecological notes for them.

## 2. Materials and Methods

### 2.1. Specimen Collections

Specimens were collected from Qipanshan Forest Park, Shenyang City; Wangbin Township, Shenyang City; and Shenxiangu, Yongling Town, Xinbin County, Liaoning Province, northeastern China. For each collection, data on specimen locality, associated vegetation, ecological habit, collector, and collection date were recorded. When multiple specimens of the same species were collected from the same general area on the same date, their collecting points were separated by at least several hundred meters. Photographs of the basidiomata and their habitats were taken in the field. The specimens were subsequently dried without delay and preserved in sealed bags. All examined specimens were deposited in the herbarium of the Institute of Applied Ecology, Chinese Academy of Sciences (IFP).

### 2.2. Morphological Studies

Macromorphological characters were observed under a stereo microscope (Nikon SMZ 645, Tokyo, Japan), and colour terms for basidiomata follow Kornerup and Wanscher [22]. Micromorphological characters were examined at 1000× magnification using a light microscope (Nikon Eclipse 80i, Tokyo, Japan). Hand-cut sections were mounted in Cotton Blue (CB), Melzer’s reagent (IKI), and 3% KOH. Line drawings of microscopic structures were made with the aid of a drawing tube. CB was used to determine cyanophily of the cell walls [23,24], IKI to test amyloid, inamyloid or dextrinoid reactions, and KOH to clear tissues, reveal pigments, and facilitate observation of tissue structures. Basidiospore length and width were measured excluding the apiculus, following common practice in fungal taxonomy. The following abbreviations are used: *L* = mean spore length, *W* = mean spore width, *Q* = *L/W* ratio, and *n* = number of basidiospores measured from a given number of specimens.

### 2.3. DNA Extraction, Amplification, and Sequencing

Total genomic DNA was extracted from dried specimens using the Rapid Fungal Genomic DNA Isolation Kit (Demeter Biotech Co., Ltd., Beijing, China) following the manufacturer’s instructions. The nuclear ribosomal internal transcribed spacer (ITS) region was amplified by polymerase chain reaction (PCR) using the primer pair ITS1 (5′-TCCGTAGGTGAACCTGCGG-3′) and ITS4 (5′-TCCTCCGCTTATTGATATGC-3′) [25,26]. The PCR program consisted of an initial denaturation at 94 °C for 5 min, followed by 34 cycles of 95 °C for 35 s, 55 °C for 55 s, and 72 °C for 45 s, with a final extension at 72 °C for 10 min. To further assess the phylogenetic placement of the collections, the nuclear ribosomal large subunit (LSU) region was amplified and sequenced using the primers LR0R (5′-ACCCGCTGAACTTAAGC-3′) and LR7 (5′-TACTACCACCAAGATCT-3′). The PCR program for LSU consisted of an initial denaturation at 94 °C for 2 min, followed by 35 cycles of 94 °C for 30 s, 48 °C for 1 min, and 72 °C for 1.5 min, with a final extension at 72 °C for 10 min [27]. PCR products were sequenced by the Beijing Genomics Institute (BGI). All newly generated sequences were assembled and manually edited in SeqMan v.7.1.0. Sequence similarity searches were conducted against GenBank using the web-based NCBI BLASTn program (National Center for Biotechnology Information, Bethesda, MD, USA; accessed on 10 March 2026) [28]. After base-calling quality was checked, the new sequences were submitted to GenBank.

### 2.4. Phylogenetic Analyses

Sequences used for phylogenetic comparisons were selected from GenBank based on BLAST similarity, taxonomic relevance, availability of voucher or specimen information, and representation of major lineages within each genus or allied group. Whenever available, sequences derived from type materials and reliably identified voucher specimens were given priority. The newly obtained sequences and selected GenBank sequences were aligned using MAFFT v.7 [29] (Table 1). All sequences were checked and adjusted to the same orientation prior to alignment, and the resulting alignments were manually refined in MEGA v.7.0 [30]. For the *Descolea* ITS + LSU combined dataset, *Hebeloma plesiocistum* and *H. theobrominum* were selected as outgroups following Kuhar et al. [2]. For the *Leucocoprinus* combined dataset, *Agaricus bisporus* was selected as the outgroup following Asif et al. [9]. For the *Tephrocybe* combined dataset, *Rhizocybe alba* and *R. vermicularis* were selected as outgroups following Khan et al. [31]. For each genus, concatenated alignments were generated from the combined ITS and LSU datasets and partitioned into four regions: ITS1, 5.8S, ITS2, and nLSU. The best-fit substitution model for each partition was selected using IQ-TREE v.2.4.0. For *Descolea*, the selected models were K2P+G4 for ITS1, JC for 5.8S, TPM2+G4 for ITS2, and K2P for nLSU. For *Leucocoprinus*, the selected models were TPM2u+F+I+G4 for ITS1, TIM2e+R2 for 5.8S, TVM+F+G4 for ITS2, and TIM3e+I+R2 for nLSU. For *Tephrocybe*, the selected models were HKY+F+G4 for ITS1, K2P+I for 5.8S, TPM2u+F+G4 for ITS2, and TN+F for nLSU. Phylogenetic analyses were performed for each dataset using Bayesian inference (BI) and maximum likelihood (ML) methods. BI analyses were conducted in MrBayes v.3.0 using a Markov chain Monte Carlo (MCMC) algorithm. Four chains were run from random starting trees for 10 million generations, with trees sampled every 1000 generations, until the average standard deviation of split frequencies fell below 0.01 [32]. The first 25% of sampled trees were discarded as burn-in, and the remaining trees were used to calculate Bayesian posterior probabilities (BPPs). ML analyses were performed using the same datasets in RAxML-HPC BlackBox v.8.2.12, and branch support was assessed with 1,000 non-parametric bootstrap replicates [33]. Phylogenetic trees were visualized using FigTree v.1.4.4 [34]. Branches with ML bootstrap support (MLBS) ≥ 70% and/or BPPs ≥ 0.95 were considered significantly supported. The alignments and trees were deposited in TreeBASE (No. S32652).

## 3. Results

### 3.1. Phylogeny

BLAST searches of the newly generated ITS sequences recovered several close GenBank matches, including unpublished, directly submitted, isolate-derived, and environmental sequences. These results indicate that the corresponding lineages, or closely related lineages, may have previously been detected in GenBank. However, BLAST parameters were used only to identify closely matching sequences and were not treated as evidence of conspecificity by themselves. Because these accessions lack formal taxonomic treatment and/or associated basidiomatal morphological data, they were not used as primary evidence for species delimitation.

The combined ITS and LSU dataset was used to infer the phylogenetic positions of the three new species. Because these taxa belong to different genera, three separate phylogenetic trees were reconstructed. The *Descolea* dataset comprised 1476 characters, including 602 from ITS and 874 from LSU. Of these, 78 were constant, 1195 were parsimony-uninformative variable, and 203 were parsimony-informative. The dataset included two sequences from the new species, 28 sequences of *Descolea* [36,37,38,39,40], and two outgroup sequences, viz. *H. plesiocistum* and *H. theobrominum* [41,42]. The *Leucocoprinus* dataset comprised 1539 characters, including 729 from ITS and 810 from LSU. Of these, 841 were constant, 204 were parsimony-uninformative variable, and 494 were parsimony-informative. The dataset included two sequences from the new species, 102 sequences of *Leucocoprinus* [43,44,45,46,47,48,49,50,51,52,53,54,55,56,57,58,59,60,61,62,63,64,65,66,67,68,69,70,71,72,73,74,75,76,77,78], and two sequences of *A. bisporus* as the outgroup [35]. Because the generic limits of *Leucocoprinus* have recently undergone substantial revision, a conservative treatment was adopted for the *Leucocoprinus* dataset. The dataset included sequences identified or formally treated as *Leucocoprinus* in GenBank and the cited taxonomic studies, because BLAST searches placed the new species among *Leucocoprinus* sequences and the collections showed morphological affinity with the traditional concept of the genus. This treatment was used to evaluate the phylogenetic placement of the new species and does not represent a broader revision of *Leucocoprinus*, *Leucoagaricus*, or allied genera. The *Tephrocybe* dataset comprised 1490 characters, including 629 from ITS and 861 from LSU. Of these, 298 were constant, 945 were parsimony-uninformative variable, and 247 were parsimony-informative. The dataset included two sequences from the new species, 21 sequences of *Tephrocybe* [81,82,83], and two outgroup sequences, *R. alba* and *R. vermicularis* [79,80]. A 50% majority-rule consensus phylogram was generated.

**Figure 1:**
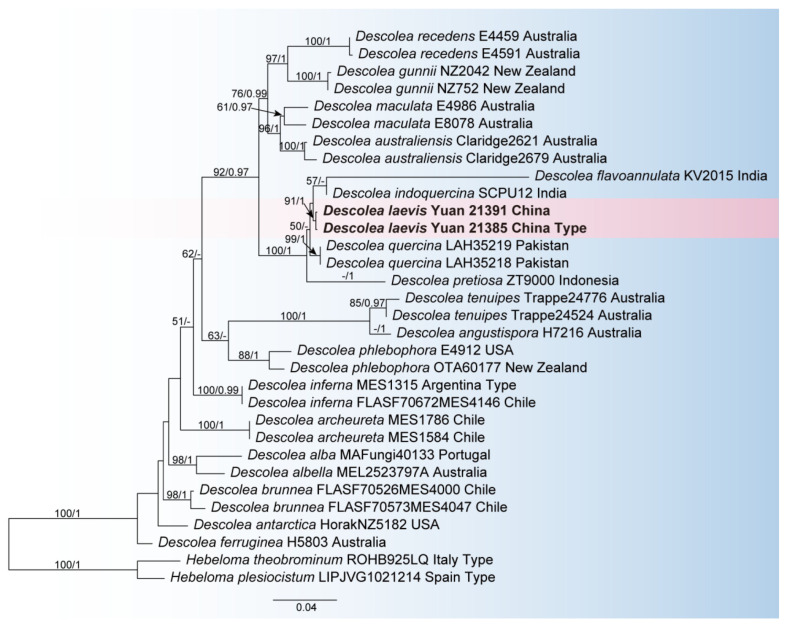
Maximum likelihood tree illustrating the phylogeny of *Descolea* and related taxa based on the combined ITS and LSU nuclear rDNA sequences dataset. Branches are labeled with maximum likelihood bootstrap values ≥ 50% and Bayesian posterior probabilities ≥ 0.95; lower values are indicated by “-”. Specimen numbers and countries of collection are indicated after species names. New species in bold (black).

**Figure 2:**
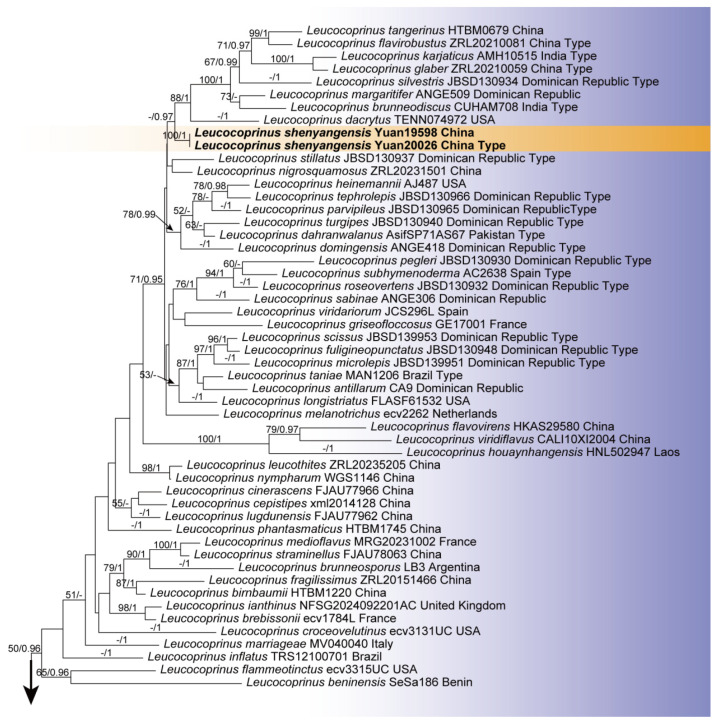

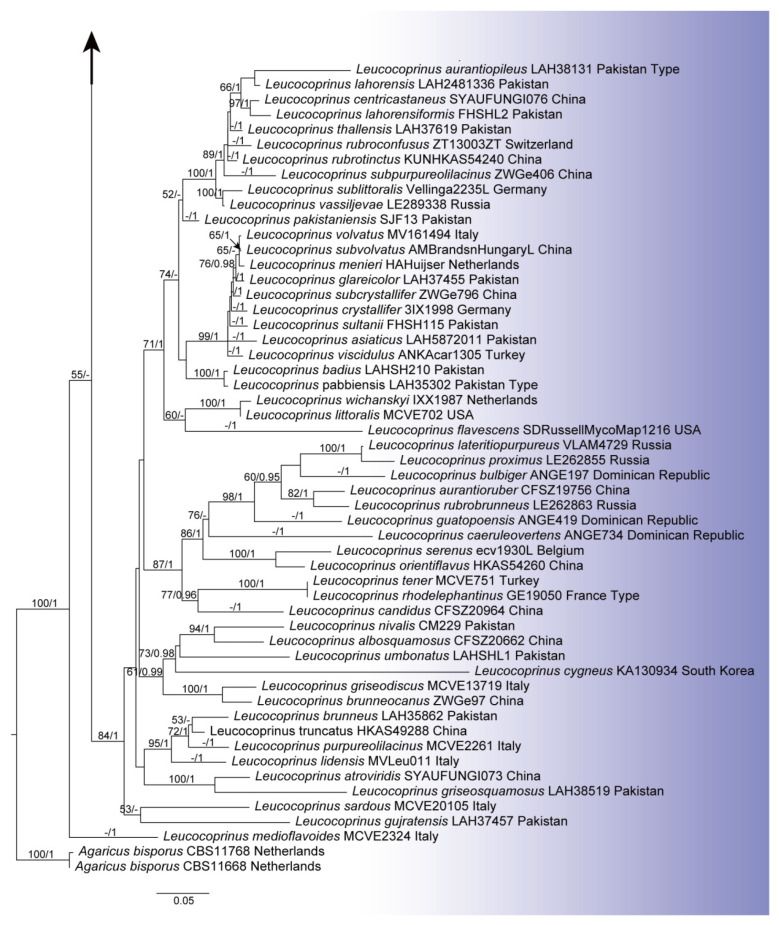
Maximum likelihood tree illustrating the phylogeny of *Leucocoprinus* and related taxa based on the combined ITS and LSU nuclear rDNA sequences dataset. Branches are labeled with maximum likelihood bootstrap values ≥ 50% and Bayesian posterior probabilities ≥ 0.95; lower values are indicated by “-”. Specimen numbers and countries of collection are indicated after species names. New species in bold (black).

**Figure 3:**
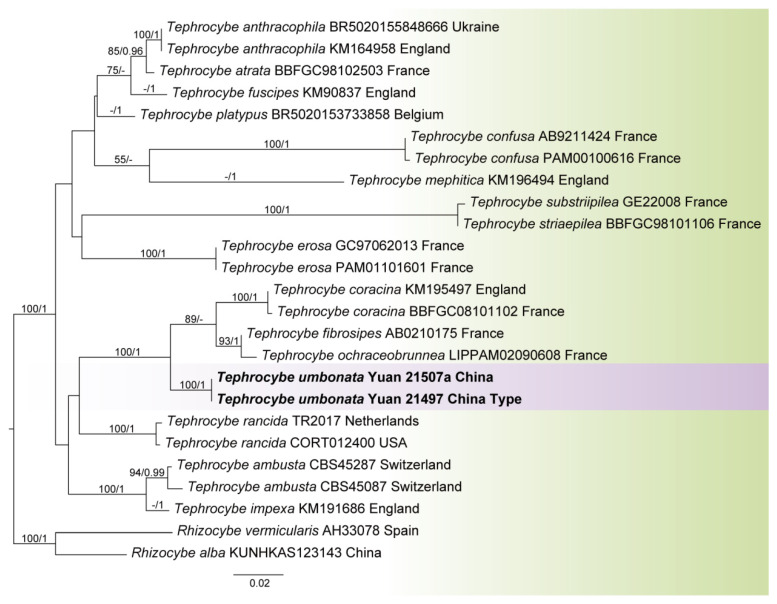
Maximum likelihood tree illustrating the phylogeny of *Tephrocybe* and related taxa based on the combined ITS and LSU nuclear rDNA sequences dataset. Branches are labeled with maximum likelihood bootstrap values ≥ 50% and Bayesian posterior probabilities ≥ 0.95; lower values are indicated by “-”. Specimen numbers and countries of collection are indicated after species names. New species in bold (black).

Maximum likelihood and Bayesian analyses recovered similar topologies; therefore, only the ML trees are presented, with MLBS and BPPs shown at the nodes. In the phylogenetic tree inferred from the combined ITS and LSU dataset, the two collections of *D. laevis* formed a distinct lineage with strong support (ML = 91%, BPP = 1.00), and this lineage was grouped with *D. flavoannulata* and *D. indoquercina* (Figure 1). The two collections of *L. shenyangensis* formed a distinct lineage with full support (ML = 100%, BPP = 1.00) (Figure 2). The two collections of *T. umbonata* also formed a distinct, fully supported lineage (ML = 100%, BPP = 1.00), which was resolved as sister to the clade comprising *T. coracina*, *T. fibrosipes*, and *T. ochraceobrunnea* (ML = 100%, BPP = 1.00) (Figure 3). These results supported the taxonomic placement of the three new species.

### 3.2. Taxonomy

**Figure 4:**
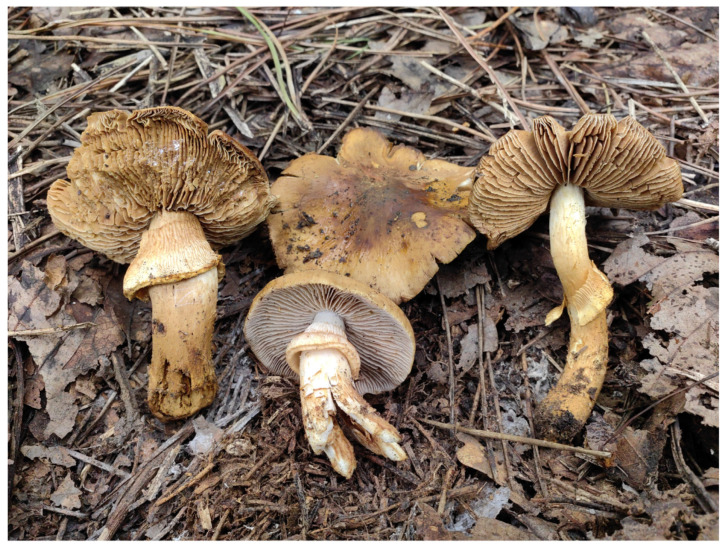
Fresh basidiomata of *Descolea laevis* (holotype, IFP 020246; specimen no. *Yuan 21,385*). Photos by Hai-Sheng Yuan.

**Figure 5:**
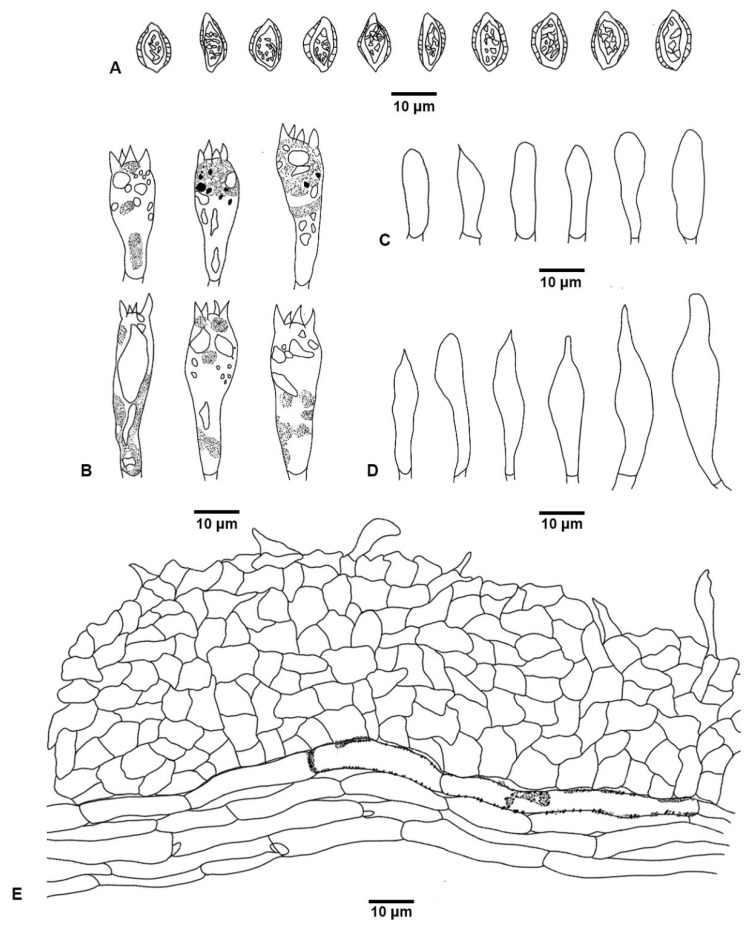
Microscopic features of *Descolea laevis* (drawn from holotype, IFP 020246; specimen no. *Yuan 21,385*) (**A**) Basidiospores (**B**) Basidia (**C**) Cheilocystidia (**D**) Pleurocystidia (**E**) Pileipellis elements. Scale bars: 10 μm.

*Descolea laevis* Z.Q. You & H.S. Yuan, sp. nov. (Figure 4 and Figure 5).

Fungal Names: FN 573717.

Diagnosis. Similar to *Descolea indoquercina*, but distinguished by smaller basidiospores (10.7–12.4 × 6.5–8.5 μm), shorter basidia (24.8–45.2 × 8.1–12.3 μm), the presence of cheilocystidia and pleurocystidia, a smooth pileus lacking scales or warts, and a thick, immovable annulus.

Type. CHINA. Liaoning Province, Shenyang City, Qipanshan Forest Park, on the ground in mixed *Quercus mongolica*-*Pinus koraiensis* forests, 18 September 2024, *Yuan 21,385* (holotype IFP 020246).

Etymology. *laevis* (Lat.), referring to the nearly smooth pileus surface lacking scales or warts.

Description. Basidiomes: medium-sized, solitary to scattered. Pileus: convex to plano-convex when young, becoming plane with a shallow central depression at maturity; surface nearly smooth, lacking scales or warts; disc greyish brown to reddish brown (9D4–9D5), margin pale yellow to light yellow (4A3–4A4); margin incurved when young, becoming straight to slightly upturned at maturity, with conspicuous irregular striations; when dry, pileus markedly shrunken, at first with a brown to dark brown centre (6E5–6F5) and a yellowish brown to linoleum brown surface (5E7–5E8), later with a greyish brown to dark brown centre (7F3–7F4) and a light brown to golden brown margin (5D6–5D7). Context: thin, slightly thicker at the centre, light orange to greyish orange (5A4–5B4). Lamellae: adnexed to adnate, rather crowded (≥14 *L* + *l*/cm), relatively broad, with lamellulae; edge uneven and slightly undulate, reddish white to pinkish white (7A2–8A2); when dry, distinctly undulate, wrinkled, and twisted, becoming tan to cognac brown (6E6–6E7). Stipe: central, cylindrical, slightly enlarged towards the base; surface dry, with conspicuous longitudinal fibrils; upper part pale, yellowish white to pale yellow (4A2–4A3), lower part light orange (5A4–5A5); basal mycelium white. Annulus: rather thick, immovable, median to superior, persistent, slightly flaring, pale yellow to pastel yellow (3A3–3A4). Odour: mild, not distinctive.

Basidiospores: (10.1–)10.7–12.4(–13.3) × (3.8–)6.5–8.5(–9.2) μm, *L* = 11.35 μm, *W* = 7.21 μm, *Q* = 1.25–1.85 (*n* = 60/2); amygdaliform to limoniform, guttulate, verrucose, slightly thick-walled, with a prominent papilla and a broad, smooth apiculus, dextrinoid, acyanophilous.

Basidia: 24.8–45.2 × 8.1–12.3 μm, narrowly clavate to subcylindrical, hyaline, thin-walled, 4-sterigmate; sterigmata slender, 3.8–6.1 × 0.8–2.2 μm; contents granular to guttulate; basal septum simple.

Cheilocystidia: 18.7–34.2 × 4.8–7.3 μm, clavate to narrowly clavate, sometimes subcylindrical, thin-walled.

Pleurocystidia: 27.6–45.3 × 4.8–12.2 μm, fusiform to narrowly fusiform, sometimes clavate, apex occasionally attenuated, thin-walled.

Pileipellis: epicutis epithelium-like, 60–166 µm thick, composed of inflated, fusiform to subglobose elements, 7.8–19.1 × 3.7–11.2 µm, surface heavily encrusted with rusty-brown pigment; subcutis composed of cylindrical, thin-walled hyphae, 2.8–10.3 µm wide, with encrusting pigment; clamp connections present.

Annulus: composed of cylindrical, smooth, hyaline, thin-walled, interwoven hyphae, 1–12 μm wide, with clamp connections.

Additional specimen (paratype) examined. CHINA. Liaoning Province, Shenyang City, Qipanshan Forest Park, on the ground in mixed *Quercus mongolica*-*Pinus koraiensis* forests, 18 September 2024, *Yuan 21,391* (IFP 020247).

**Figure 6:**
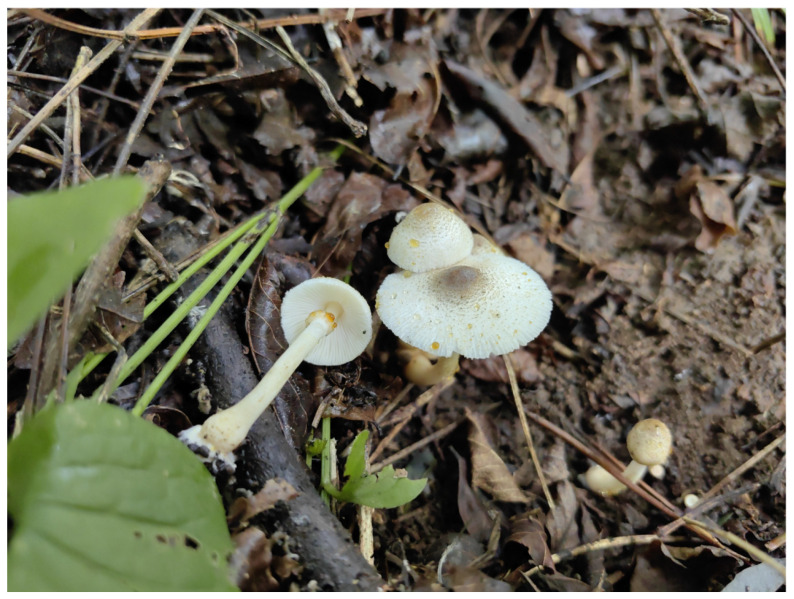
Fresh basidiomata of *Leucocoprinus shenyangensis* (holotype, IFP 020248; specimen no. *Yuan 20,026*). Photos by Hai-Sheng Yuan.

**Figure 7:**
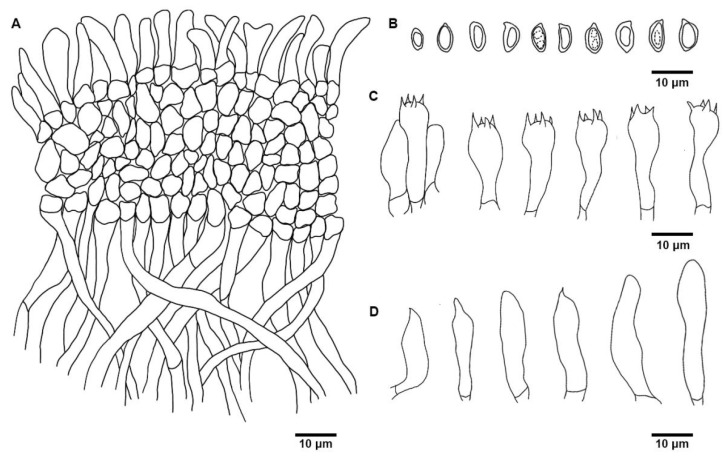
Microscopic features of *Leucocoprinus shenyangensis* (drawn from holotype, IFP 020248; specimen no. *Yuan 20,026*) (**A**) Pileipellis elements (**B**) Basidiospores (**C**) Basidia (**D**) Cheilocystidia. Scale bars: 10 μm.

*Leucocoprinus shenyangensis* Z.Q. You & H.S. Yuan, sp. nov. (Figure 6 and Figure 7).

Fungal Names: FN 573718.

Diagnosis. Morphologically similar to *Leucocoprinus dacrytus,* but distinguished by basidiospores lacking a germ pore, thin-walled cheilocystidia, and a hymenidermal epicutis of the pileipellis composed of subglobose to short ellipsoid cells with narrowly clavate to clavate terminal elements, gradually passing into an interwoven hyphal layer below.

Type. CHINA. Liaoning Province, Shenyang City, Wangbin Township, on the ground in mixed forests, 6 August 2024, *Yuan 20,026* (holotype IFP 020248).

Etymology. *shenyangensis* (Lat.), named after the collection site of the type specimen, Shenyang City.

Description. Basidiomes: small, solitary to scattered. Pileus: ovoid-campanulate to campanulate when young, expanding to convex to nearly plane, with a low but distinct obtuse umbo at the centre; surface exuding droplets of various sizes, light yellow to orange (4A4–5A7), scattered with minute light grey (8C1–8D1) granulose to squamulose elements; disc greyish brown to reddish brown (8D3–8D4), margin paler, white to yellowish white (3A1–3A2); margin distinctly striate at maturity, the striations extending over much of the pileus radius; when dry, pileus markedly shrunken and radially plicate-striate, with the disc darker, sunburn to tan brown (6D5–6E5), and the margin yellowish white to pale yellow (4A2–4A3). Context: thin, white to orange white (5A1–5A2). Lamellae: free, rather crowded, subequal in length, with entire edges, white to yellowish white (1A1–1A2); when dry, slightly crisped, light yellow to champagne yellow (4A4–4B4). Stipe: central, cylindrical, slender, slightly enlarged towards the base; surface smooth to finely fibrillose, smoother above the annulus; lower part pale yellow, becoming white upwards (4A3–4A1); basal mycelium white. Annulus: thin, membranous, median to superior, persistent, orange white to pale orange (5A2–5A3); surface sometimes exuding light yellow to orange (4A4–5A7) droplets. Odour: not recorded.

Basidiospores: (3.1–)3.5–7.2(–9.0) × (2.5–)2.8–4.5(–5.5) μm, *L* = 6.14 μm, *W* = 3.79 μm, *Q* = 1.01–2.40 (*n* = 60/2); ovoid to ellipsoid-oblong in frontal view, amygdaliform to limoniform in side view, smooth, hyaline, without a distinct germ pore, slightly thick-walled, dextrinoid, cyanophilous.

Basidia: 15.8–26.3 × 6.7–9.2 μm, narrowly clavate to subcylindrical, hyaline, thin-walled, usually 4-sterigmate; basal septum simple.

Cheilocystidia: 23.2–36.8 × 4.8–8.3 μm, narrowly clavate to subcylindrical, often slightly curved, apex rounded to slightly acute, base gradually narrowed, hyaline, thin-walled.

Pleurocystidia: absent.

Pileipellis: epicutis hymenidermal, composed of rather regularly arranged, subglobose to short ellipsoid cells, with terminal elements narrowly clavate to clavate, suberect to erect, (14.2–)17.1–19.8(–22.9) × (2.7–)5.1–6.2(–8.4) μm, apex rounded to slightly acute, sometimes containing pale brown to brown intracellular pigment; subcutis composed of interwoven, subcylindrical, smooth, hyaline, slightly thick-walled hyphae, 2–10 μm wide, gradually passing into the context; clamp connections not observed.

Annulus: composed of cylindrical, smooth, hyaline, occasionally branched hyphae, 1.2–13.5 µm wide, with occasional rusty brown to reddish-brown intracellular pigment, without clamp connections.

Additional specimens (paratype) examined. CHINA. Liaoning Province, Shenyang City, Qipanshan Forest Park, on the ground in *Populus* forest, 6 August 2024, *Yuan 19,598* (IFP 020249).

**Figure 8:**
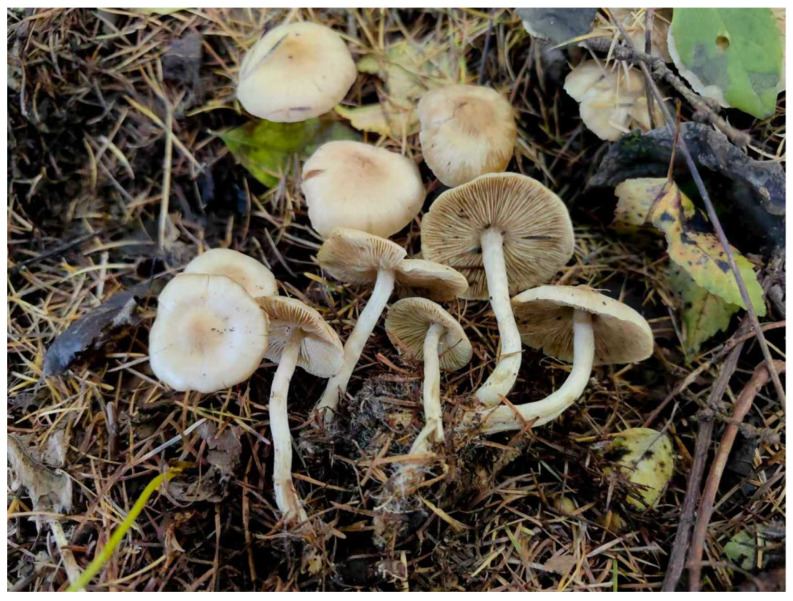
Fresh basidiomata of *Tephrocybe umbonata* (holotype, IFP 020250; specimen no. *Yuan 21,497*). Photos by Hai-Sheng Yuan.

**Figure 9:**
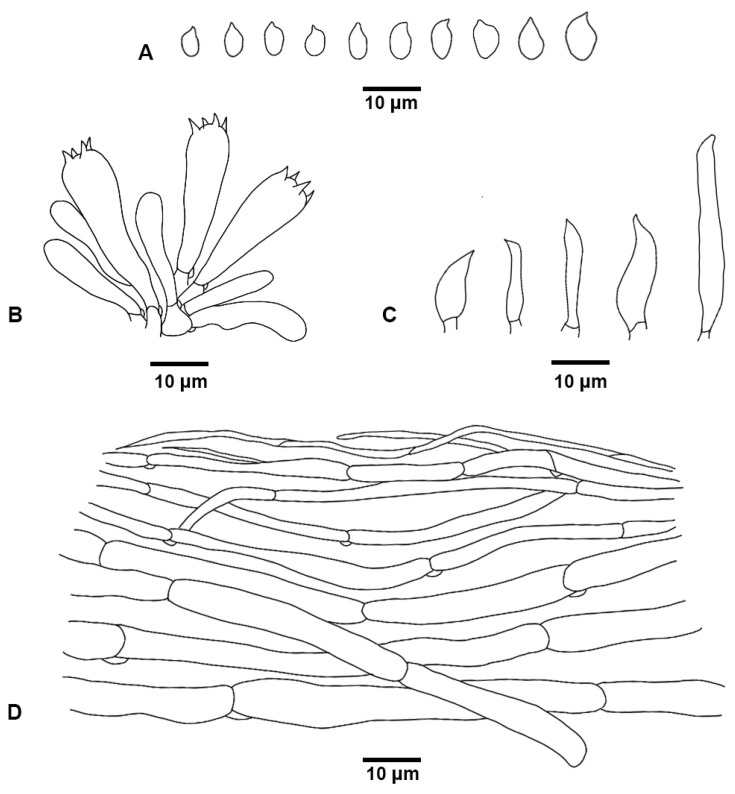
Microscopic features of *Tephrocybe umbonata* (drawn from holotype, IFP 020250; specimen no. *Yuan 21,497*) (**A**) Basidiospores (**B**) Basidia and Marginal cells (**C**) Pleurocystidia (**D**) Pileipellis elements. Scale bars: 10 μm.

*Tephrocybe umbonata* Z.Q. You & H.S. Yuan, sp. nov. (Figure 8 and Figure 9).

Fungal Names: FN 573719.

Diagnosis. Similar to *Tephrocybe platypus*, but distinguished by a distinct obtuse umbo at the centre of the pileus, basidia usually 4-spored, and pleurocystidia with slightly acute apices.

Type. CHINA. Liaoning Province, Xinbin County, Yongling Town, Shenxiangu, on the ground in *Larix kaempferi* forest, 4 October 2024, *Yuan 21,497* (holotype IFP 020250).

Etymology. *umbonata* (Lat.), referring to the distinct umbo at the centre of the pileus.

Description. Basidiomes: small, solitary to subgregarious. Pileus: hemispherical to convex when young, becoming nearly plane with a distinct obtuse umbo to low papilla at the centre; surface dry, smooth or slightly finely fibrillose; disc reddish white to reddish grey (8A2–8B2), margin paler, orange white to greyish orange (5A2–5B4); margin with conspicuous wavy translucent striations, sometimes shallowly cracked; when dry, pileus shrunken, with a distinct central depression and an oak brown to linoleum brown (5D6–5E7) surface. Context: thin, white to reddish white (7A1–7A2). Lamellae: rather distant to moderately crowded, with nearly entire edges, orange white to pale orange (5A2–5A3); when dry, becoming dark blonde to mustard brown (5D4–5E6). Stipe: central, subcylindrical, slender, often curved, fibrous; surface smooth at the base, slightly roughened and covered with white granulose remnants towards the apex, white to pale yellow (4A1–4A3); basal mycelium white. Annulus: absent. Odour: not recorded.

Basidiospores: (4.2–)4.7–7.5(–8.1) × (2.0–)2.8–4.1(–4.3) μm, *L* = 6.64 μm, *W* = 3.49 μm, *Q* = 1.13–2.67 (*n* = 60/2); ellipsoid to ovoid in frontal view, amygdaliform to limoniform in side view, smooth, hyaline, guttulate, moderately thick-walled, with a distinct lateral apiculus, inamyloid, cyanophilous.

Basidia: 19.8–31.2 × 4.9–9.3 μm, clavate to narrowly clavate, rarely subcylindrical, slightly curved, hyaline, thin-walled, 4-sterigmate; contents granular, non-metachromatic; basal clamp connections present.

Cheilocystidia: absent.

Pleurocystidia: 14.7–39.3 × 2.0–5.1 μm, lageniform to narrowly lageniform, sometimes fusiform, rarely subcylindrical, apex slightly acute, base gradually narrowed, thin-walled.

Marginal cells: 16.7–27.3 × 3.8–7.2 μm, resembling pseudocystidia, narrowly clavate to cylindrical, sometimes elongated-cylindrical, occasionally irregularly curved.

Pileipellis: epicutis a non-gelatinized cutis, rather compact, composed of slender, repent, parallel to subparallel hyphae, 0.5–5.1 μm wide, locally slightly interwoven, without evident erect terminal elements; subcutis indistinct, composed of loosely arranged hyphae, 4.2–12.3 μm wide, gradually passing into the context; clamp connections present.

Annulus: absent.

Additional specimens (paratype) examined. CHINA. Liaoning Province, Xinbin County, Yongling Town, Shenxiangu, on the ground in *Larix kaempferi* forest, 4 October 2024, *Yuan 21,507a* (IFP 020251).

## 4. Discussion

In this study, three new species belonging to *Descolea*, *Leucocoprinus*, and *Tephrocybe* were confirmed from the areas surrounding Shenyang, Liaoning Province, China, based on morphological observations and molecular phylogenetic analyses. Phylogenetic results support these taxa as independent lineages within their respective genera, and morphological comparisons further show that they can be clearly distinguished from their allied species.

In the phylogenetic tree, the two collections of *Descolea laevis* were placed within a clade including *D. flavoannulata*, *D. indoquercina*, *D. quercina*, and *D. pretiosa* (ML = 100%, BPP = 1.00). These species share annulate basidiomata, amygdaliform to limoniform basidiospores bearing verrucose ornamentation, a conspicuous papilla, and a smooth apiculus. *D. laevis* differs from *D. indoquercina* by its smaller basidiospores and the presence of both pleurocystidia and cheilocystidia. *D. laevis* differs from *D. flavoannulata* in possessing pleurocystidia and a thick, immovable annulus, although the basidia are similar in size [84]. *D. quercina* is distinguished by more coarsely verrucose basidiospores and by having 2-spored basidia with clamp connections at the base [3]. *D. pretiosa* differs from *D. laevis* in having a pileus densely covered with ochraceous brown to pale ochraceous yellow scales, lacking cheilocystidia, and having an epithelium of clavate cells in the pileipellis [1].

*Leucocoprinus shenyangensis* is readily recognized by its slender stipe, overall pale yellow to white basidiomata, and pileus with a greyish brown to reddish brown blunt umbo. Phylogenetically, *L. shenyangensis* formed a distinct and fully supported lineage in the combined ITS and LSU analyses. It was placed within a well-supported clade including *L. dacrytus*, *L. brunneodiscus*, *L. margaritifer*, *L. silvestris*, *L. glaber*, *L. karjaticus*, *L. flavirobustus*, and *L. tangerinus* (BPP = 0.97), with *L. dacrytus* recovered as its closest relative. The two species are similar in basidiospore size and shape, but *L. dacrytus* differs in having slightly larger, thick-walled cheilocystidia and a cutis-like pileus covering [80]. *L. brunneodiscus* differs in its orange basidiomata, 2-spored basidia, clavate cheilocystidia, and subcutis- to cutis-like pileus covering [49]. *L. margaritifer* is distinguished by its brownish pink pileus, larger, branched cheilocystidia, and trichodermal pileus covering [43]. *L. silvestris* differs in having rosy to pink-ocher squamules on the pileus, a pinkish stipe base, branched cheilocystidia, and a trichodermal pileus covering [43]. *L. glaber*, *L. karjaticus*, and *L. flavirobustus* form a small allied subclade, and share 2-spored basidia, cylindrical to capitate cheilocystidia composed of cell chains, and a pileus covering composed of cylindrical element chains. However, *L. glaber* has a light orange pileus margin [52], *L. karjaticus* has larger basidiomata and yellowish brown to reddish brown squamules [65]. *L. flavirobustus* has a darker reddish brown pileus centre [52]. *L. tangerinus* is readily distinguished by the presence of pleurocystidia [85]. The exudation of colored droplets is not unique to *L. shenyangensis*, as similar golden yellow to brown, orange-white, or amber droplets have also been reported in some allied taxa, including *L. dacrytus*, *L. brunneodiscus*, and *L. margaritifer* [43,49,80]. However, taxa reported to exude colored droplets were not recovered as a separate, well-supported monophyletic group in the present ITS and LSU phylogeny, but were placed within a broader clade that also included species with colorless droplets or without reported droplet exudation. Thus, this feature is not interpreted as evidence for an independent infrageneric lineage.

*Tephrocybe umbonata* formed a fully supported clade with *T. ochraceobrunnea*, *T. fibrosipes*, and *T. coracina*, which was sister to *T. rancida*. These allied taxa share several characters, including 4-spored basidia with conspicuous siderophilous granules, absence of cheilocystidia, and a pileipellis composed of filamentous hyphae. Nevertheless, *T. umbonata* differs from the other similar species in having pleurocystidia with slightly pointed apices. Although *T. ochraceobrunnea* and *T. fibrosipes* resemble *T. umbonata* in their relatively pale basidiomata, *T. ochraceobrunnea* has broader basidiospores (4.0–5.0 μm wide), whereas *T. fibrosipes* has larger basidiospores (7–9 × 4–5 μm) [86,87]. By contrast, *T. coracina* and *T. rancida* have darker basidiomata, from deep sooty-brown to nearly black. *T. umbonata* further differs from *T. coracina* in having narrower basidiospores and from *T. rancida* in lacking a pseudorrhiza at the stipe base [88].

The three new species were collected from distinct forest-floor habitats around Shenyang, including mixed *Quercus mongolica*-*Pinus koraiensis* forests, *Populus* forests or mixed forests, and *Larix kaempferi* forests. *Descolea laevis* was collected on the ground in a mixed forest of *Q*. *mongolica* and *P*. *koraiensis*. BLAST results suggest that this species, or a closely related lineage, may have been detected previously in forest soils from Anhui Province, China (GenBank accession no. PX601745), and from Changbai Mountain, Jilin Province, China (e.g., GenBank accession no. JQ666800). *D. indoquercina*, *D. flavoannulata*, and *D. quercina* have all been reported from Himalayan forests dominated by *Quercus* spp. [84]. This pattern suggests that closely related taxa of *Descolea* may occur across a relatively broad range of forest habitats, including *Quercus*-dominated and mixed forests. *Leucocoprinus shenyangensis* and its allied taxa are saprotrophic and mainly occur on the forest floor and other organic-rich substrates [43,65,85]. BLAST results indicate that this species, or a closely related lineage, may be more widely distributed in China (e.g., GenBank accession no. PX513137). *L. shenyangensis* was collected on the ground in a *Populus* and a mixed forest, whereas *L. dacrytus* occurs on decayed wood of *Quercus rubra* in deciduous forest [89], indicating differentiation in substrate use despite a shared forest habitat background. Lyophyllaceae includes ecologically diverse taxa, and multigene phylogenetic studies have suggested repeated transitions from free-living saprotrophic to parasitic or mutualistic lifestyles within the family [19]. Several lyophylloid taxa occupy specialized substrates, including pyrophilous habitats, as shown by *Tephrocybe anthracophila* among fungi fruiting after fire [90], and nitrogen-rich substrates, as shown by the ammonia fungus *T. tesquorum* in urea-treated forest soil [91]. A blue reaction with p-dimethylaminobenzaldehyde (PDAB) has also been discussed as a macroscopic chemical character in lyophylloid fungi [92]. In contrast to these specialized ecological patterns, several species of *Tephrocybe* have been reported from relatively general forest-floor habitats, including soil, humus, litter layers, and adjacent woodland ground [21,88]. *T. umbonata* was collected on the ground in a *Larix kaempferi* forest. BLAST results suggest that this species, or a closely related lineage, may have been detected previously in Virginia, USA (e.g., GenBank accession no. PX925887), and in beech-maple forests from northeastern Ohio, USA (GenBank accession no. FM999645) [93]. By contrast, *T. rancida* is more common in deciduous litter, especially in *Fagus* forests, and only occasionally occurs in coniferous forests [88]. Together, these habitat records place the three new species in a more specific regional and ecological context and support continued targeted taxonomic surveys of macrofungi in Shenyang and adjacent areas.

## Figures and Tables

**Table 1:** Species and GenBank numbers used in phylogenetic analysis in this study.

Species Name	ITS	LSU	Specimen No.	Country	References
*Agaricus bisporus* (J.E. Lange) Imbach	MH859080	MH870797	CBS11668	The Netherlands	Vu et al., 2019 [35]
*A. bisporus*	MH859081	MH870798	CBS11768	The Netherlands	Vu et al., 2019 [35]
*Descolea alba* (Bull.) Kuhar, Nouhra & M.E. Sm.	AJ296296	/	MA-Fungi40133	Portugal	Martín and Moreno 2001 [36]
*D. albella* (Massee & Rodway) Kuhar, Nouhra & M.E. Sm.	PV650409	/	MEL2523797A	Australia	NCBI Database
*D. angustispora* (A.A. Francis & Bougher) Kuhar, Nouhra & M.E. Sm.	DQ328058	/	H7216	Australia	Francis and Bougher 2004 [37]
*D. antarctica* Singer	AF325647	/	HorakNZ5182	New Zealand	Peintner et al., 2001 [38]
*D. archeureta* (Halling) Kuhar, Nouhra & M.E. Sm.	KY523096	/	MES-1786	Chile	Kuhar et al., 2017 [2]
*D. archeureta*	KY523092	/	MES-1584	Chile	Kuhar et al., 2017 [2]
*D. australiensis* (G.W. Beaton, Pegler & T.W.K. Young) Kuhar, Nouhra & M.E. Sm.	AF325627	/	Claridge2679	Australia	Peintner et al., 2001 [38]
*D. australiensis*	AF325628	/	Claridge2621	Australia	Peintner et al., 2001 [38]
*D. brunnea* (E. Horak) Kuhar, Nouhra & M.E. Sm.	OP339609	/	FLAS:F-70573-MES-4047	Chile	NCBI Database
*D. brunnea*	OP339580	/	FLAS:F-70526-MES-4000	Chile	NCBI Database
*D. ferruginea* (J.W. Cribb) Kuhar, Nouhra & M.E. Sm.	DQ328083	/	H5803	Australia	Kuhar et al., 2017 [2]
*D. flavoannulata* (Lj.N. Vassiljeva) E. Horak	PP345433	/	KV-20-15	India	NCBI Database
*D. gunnii* (Berk. ex Massee) E. Horak	AF325652	/	NZ752	New Zealand	Peintner et al., 2001 [38]
*D. gunnii*	AF325653	/	NZ2042	New Zealand	Peintner et al., 2001 [38]
*D. indoquercina* S. Choudhary, P. Uniyal & Y.P. Sharma	OR979479	/	SC/PU/12	India	NCBI Database
*D. inferna* Kuhar, Nouhra & M.E. Sm.	NR154032	/	CORD:MES1315 (T)	Argentina	Kuhar et al., 2017 [2]
*D. inferna*	OP339680	/	FLAS:F70672MES-4146	Chile	NCBI Database
***D. laevis*** **Z.Q. You & H.S. Yuan**	**PZ235489**	**PZ235510**	**Yuan21385 (T)**	**China**	**Present study**
***D. laevis***	**PZ235490**	**PZ235511**	**Yuan21391**	**China**	**Present study**
*D. maculata* Bougher	DQ192181	DQ457664	E8078(PERTH)	Australia	Matheny et al., 2006 [39]
*D. maculata*	AF325651	/	E4986	Australia	Peintner et al., 2001 [38]
*D. phlebophora* E. Horak	AF325657	/	E4912	Australia	Peintner et al., 2001 [38]
*D. phlebophora*	JX178627	/	OTA:60177	New Zealand	Teasdale et al., 2013 [40]
*D. pretiosa* E. Horak	MN267170	/	ZT9000	Indonesia	NCBI Database
*D. quercina* J. Khan & Naseer	MF966638	/	LAH35219	Pakistan	Khan et al., 2017 [3]
*D. quercina*	MF966637	MF966635	LAH35218	Pakistan	Khan et al., 2017 [3]
*D. recedens* (Sacc.) Singer	AF325648	/	E4591	Australia	Peintner et al., 2001 [38]
*D. recedens*	AF325649	/	E4459	Australia	Peintner et al., 2001 [38]
*D. tenuipes* (Setch.) Neville & Poumarat	AF325624	/	Trappe24776	Australia	Peintner et al., 2001 [38]
*D. tenuipes*	AF325623	/	Trappe24524	Australia	Peintner et al., 2001 [38]
*Hebeloma plesiocistum* Beker, U. Eberh. & Vila	NR119686	/	LIP:JVG1021214 (T)	Spain	Eberhardt et al., 2009 [41]
*H. theobrominum* Quadr.	NR120177	/	ROHB:925LQ (T)	Italy	Eberhardt et al., 2013 [42]
*Leucocoprinus albosquamosus* (Y.R. Ma, Z.W. Ge & T.Z. Liu) M. Asif, Saba & Vellinga	OM976879	OM976865	CFSZ20662	China	Ma et al., 2022 [8]
*L. antillarum* Justo, Bizzi & Angelini	MN482989	/	CA9	Dominican Republic	Justo et al., 2021 [43]
*L. asiaticus* (Qasim, Nawaz & Khalid) M. Asif, Saba & Vellinga	KP164972	/	LAH5872011	Pakistan	Ge et al., 2015 [44]
*L. atroviridis* (Y.R. Ma, Z.W. Ge & T.Z. Liu) M. Asif, Saba & Vellinga	OM976852	OM976868	SYAUFUNGI073	China	Ma et al., 2022 [8]
*L. aurantiopileus* Maula, Asif, A.K. Rani & Afshan	PP383877	PP583799	LAH38131 (T)	Pakistan	Maula et al., 2026 [45]
*L. aurantioruber* (Y.R. Ma, Z.W. Ge & T.Z. Liu) M. Asif, Saba & Vellinga	OM976875	OM976863	CFSZ19756	China	Ma et al., 2022 [8]
*L. badius* (S. Hussain, Pfister, Afshan & Khalid) M. Asif, Saba & Vellinga	KU647734	/	LAHSH210	Pakistan	Hussain et al., 2018 [46]
*L. beninensis* Sarawi	PX634061	/	SeSa186	Benin	Sarawi et al., 2026 [47]
*L. birnbaumii* (Corda) Singer	PQ321881	PQ319807	HTBM1220	China	Yang et al., 2024 [10]
*L. brebissonii* (Godey) Locq.	AF482859	AY176446	ecv1784L	France	Vellinga et al., 2003 [48]
*L. brunneocanus* (Fei Yu, Jun F. Liang & Z.W. Ge) M. Asif, Saba & Vellinga	KP096237	/	ZWGe97	China	Ge et al., 2015 [44]
*L. brunneodiscus* (A.K. Dutta & K. Acharya) Kun L. Yang, Jia Y. Lin & Zhu L. Yang	NR198110	NG244261	CUHAM708 (T)	India	Dutta et al., 2021 [49]
*L. brunneosporus* B.E. Lechner & J.M. Suárez	MT796198	MT796197	LB3	Argentina	Suarez et al., 2021 [50]
*L. brunneus* (Zia Ullah, Jabeen & Khalid) M. Asif, Saba & Vellinga	MH990662	/	LAH35862	Pakistan	Ullah et al., 2019 [51]
*L. bulbiger* (Justo, Bizzi & Angelini) M. Asif, Saba & Vellinga	MN483028	/	ANGE197	Dominican Republic	Justo et al., 2021 [43]
*L. caeruleovertens* (Justo, Bizzi & Angelini) M. Asif, Saba & Vellinga	MN483032	/	ANGE734	Dominican Republic	Justo et al., 2021 [43]
*L. candidus* (Y.R. Ma, Z.W. Ge & T.Z. Liu) M. Asif & Saba	OM976877	OM976864	CFSZ20964	China	Ma et al., 2022 [8]
*L. centricastaneus* (Y.R. Ma, Z.W. Ge & T.Z. Liu) M. Asif, Saba & Vellinga	OM976855	OM976871	SYAUFUNGI076	China	Ma et al., 2022 [8]
*L. cepistipes* (Sowerby) Pat.	PV470803	PV476031	xml2014128	China	Li et al., 2025 [52]
*L. cinerascens* (Quél.) Locq.	PX527069	PX527070	FJAU77966	China	NCBI Database
*L. croceovelutinus* Bon & Boiffard	EU166351	/	ecv3131UC	USA	Vellinga and Sundberg 2008 [53]
*L. crystallifer* (Vellinga) Migl. & Donato	AF482863	AY176412	3IX1998	Germany	Vellinga et al., 2003 [48]
*L. cygneus* (J.E. Lange) Bon	KR673661	/	KA130934	Republic of Korea	Kim et al., 2015 [54]
*L. dacrytus* (Vellinga) Kun L. Yang, Jia Y. Lin & Zhu L. Yang	MT196954	/	TENN:074972	USA	Swenie and Matheny 2023 [55]
*L. dahranwalanus* Asif, Saba & Raza	OQ947827	OQ947833	AsifSP71AS67 (T)	Pakistan	Asif et al., 2024 [9]
*L. domingensis* Justo, Bizzi, Angelini &Vizzini	MN483016	/	ANGE418 (T)	Dominican Republic	Justo et al., 2021 [43]
*L. flammeotinctus* (Kauffman) Redhead	GU136165	/	ecv3315UC	USA	Vellinga 2010 [56]
*L. flavescens* (Morgan) H.V. Sm.	MW567852	/	SDRussellMycoMa-p1216	USA	NCBI Database
*L. flavirobustus* R.L. Zhao & J.X. Li	PV470825	PV475985	ZRL20210081 (T)	China	Li et al., 2025 [52]
*L. flavovirens* (Jun F. Liang, Zhu L. Yang & J. Xu) Kun L. Yang, Jia Y. Lin & Zhu L. Yang	EU416293	EU416294	HKAS29580	China	Liang et al., 2010 [57]
*L. fragilissimus* (Ravenel ex Berk. & M.A. Curtis) Pat.	LT716029	KY418844	ZRL20151466	China	Zhao et al., 2017 [58]
*L. fuligineopunctatus* Justo, Bizzi & Angelini	NR173872	/	JBSD130948 (T)	Dominican Republic	Justo et al., 2021 [43]
*L. glaber* R.L. Zhao & J.X. Li	PV470824	PV475895	ZRL20210059 (T)	China	Li et al., 2025 [52]
*L. glareicolor* (S. Ashraf, Naseer & Khalid) M. Asif, Saba & Vellinga	OP605604	OP782028	LAH37455	Pakistan	Ashraf et al., 2023 [59]
*L. griseodiscus* (Bon) Migl. & Donato	GQ329059	/	MCVE13719	Italy	Osmundson et al., 2013 [60]
*L. griseofloccosus* Lagardère & Eyssart.	MH257568	/	GE17001	France	Lagardère and Eyssartier 2016 [61]
*L. griseosquamosus* (Sysouph. & Thongkl.) Kun L. Yang, Jia Y. Lin & Zhu L. Yang	PQ871415	/	LAH38519	Pakistan	NCBI Database
*L. guatopoensis* (Dennis) M. Asif, Saba & Vellinga	MN483031	/	ANGE419	Dominican Republic	Justo et al., 2021 [43]
*L. gujratensis* (A. Rehman, Usman, Afshan & Khalid) M. Asif, Saba & Vellinga	OP526420	OP885328	LAH37457	Pakistan	Rehman et al., 2023 [62]
*L. heinemannii* Migl.	MN483010	/	AJ487	USA	Justo et al., 2021 [43]
*L. houaynhangensis* (Sysouph.) Kun L. Yang, Jia Y. Lin & Zhu L. Yang	KX640915	/	HNL502947	Laos	Sysouphan-thong et al., 2018 [63]
*L. ianthinus* (Sacc.) P. Mohr	PQ796880	/	NFSG2024092201-AC	United Kingdom	NCBI Database
*L. inflatus* Raithelh.	MK685764	/	TRS12100701	Brazil	Solomon et al., 2019 [64]
*L. karjaticus* (P.B. Patil, N.P. Patil, S. Chahar & S. Maurya) R.L. Zhao & J.X. Li	NR198656	NG243935	AMH10515 (T)	India	Patil et al., 2024 [65]
*L. lahorensiformis* (S. Hussain, H. Ahmad, Afshan & Khalid) M. Asif, Saba & Vellinga	KU647730	KU900516	FHSHL2	Pakistan	Hussain et al., 2018 [46]
*L. lahorensis* (Qasim, T. Amir & Nawaz) M. Asif, Saba & Vellinga	KJ701796	/	LAH2481336	Pakistan	Qasim et al., 2015 [66]
*L. lateritiopurpureus* (Lj.N. Vassiljeva) M. Asif, Saba & Vellinga	JX133174	/	VLAM4729	Russia	Malysheva et al., 2013 [67]
*L. leucothites* (Vittad.) Redhead	PV470789	PV476017	ZRL20235205	China	Li et al., 2025 [52]
*L. lidensis* (Migl. & P. Alvarado) Migl. & Donato	MT416130	/	MVLeu011	Italy	Migliozzi and Alvarado 2021 [68]
*L. littoralis* (Ménier) M. Asif, Saba & Vellinga	GQ329041	/	MCVE702	Italy	Osmundson et al., 2013 [60]
*L. longistriatus* (Peck) H.V. Sm. & N.S. Weber	MH211956	/	FLASF61532	USA	NCBI Database
*L. lugdunensis* S. Basso & N. Schwab	PX527021	PX527063	FJAU77962	China	NCBI Database
*L. margaritifer* (Justo, Bizzi & Angelini) M. Asif, Saba & Vellinga	MN482997	/	ANGE509	Dominican Republic	Justo et al., 2021 [43]
*L. marriageae* (D.A. Reid) Migl. & Donato	PV026219	/	MV040040	Italy	Migliozzi and Donato 2025 [69]
*L. medioflavoides* (Bon) Migl. & Donato	GQ329055	/	MCVE2324	Italy	Osmundson et al., 2013 [60]
*L. medioflavus* (Boud.) Bon	PQ570805	/	MRG20231002	France	NCBI Database
*L. melanotrichus* (Malençon & Bertault) Migl. & Donato	AY176417	/	ecv2262	Netherlands	Vellinga 2004 [5]
*L. menieri* (Sacc.) Migl. & Donato	KP300879	/	HAHuijser	Netherlands	Ge et al., 2015 [44]
*L. microlepis* Justo, Bizzi & Angelini	NR173874	/	JBSD139951 (T)	Dominican Republic	Justo et al., 2021 [43]
*L. nigrosquamosus* R.L. Zhao & J.X. Li	PV470838	PV476015	ZRL20231501	China	Li et al., 2025 [52]
*L. nivalis* (W.F. Chiu) M. Asif, Saba & Vellinga	MK106151	/	CM229	Pakistan	Jabeen et al., 2020 [70]
*L. nympharum* (Kalchbr.) M. Asif, Saba & Vellinga	OM974312	OM967233	WGS1146	China	Ma et al., 2022 [8]
*L. orientiflavus* (Z.W. Ge) M. Asif, Saba & Vellinga	GU084262	JN940290	HKAS54260	China	Ge 2010 [71]
*L. pabbiensis* (Usman & Khalid) M. Asif, Saba & Vellinga	MG973423	/	LAH35302 (T)	Pakistan	Usman and Khalid 2018 [72]
*L. pakistaniensis* (Jabeen & Khalid) Asif & Saba	KU647727	KU900515	SJF13	Pakistan	Hussain et al., 2018 [46]
*L. parvipileus* Justo, Bizzi, Angelini & Vizzini	NR200351	/	JBSD130965 (T)	Dominican Republic	Justo et al., 2021 [43]
*L. pegleri* (Justo, Bizzi & Angelini) M. Asif, Saba & Vellinga	NR173878	/	JBSD130930 (T)	Dominican Republic	Justo et al., 2021 [43]
*L. phantasmaticus* Kun L. Yang, Jia Y. Lin & Zhu L. Yang	PV620895	PV616973	HTBM1745	China	Yang et al., 2025 [73]
*L. proximus* (E.F. Malysheva, Svetash. & E.M. Bulakh) M. Asif, Saba & Vellinga	JX133171	/	LE262855	Russia	Malysheva et al., 2013 [67]
*L. purpureolilacinus* (Huijsman) M. Asif, Saba & Vellinga	GQ329053	/	MCVE2261	Italy	Osmundson et al., 2013 [60]
*L. rhodelephantinus* (Boisselet & Eyssart.) Migl. & Donato	MT984270	/	GE19050 (T)	France	Boisselet and Eyssartier 2020 [74]
*L. roseovertens* (Justo, Bizzi & Angelini) M. Asif, Saba & Vellinga	NR173879	/	JBSD130932 (T)	Dominican Republic	Justo et al., 2021 [43]
*L. rubrobrunneus* (E.F. Malysheva, Svetash. & E.M. Bulakh) M. Asif, Saba & Vellinga	JX133168	/	LE262863	Russia	Malysheva et al., 2013 [67]
*L. rubroconfusus* (Migl. & Coccia) Redhead	KP300875	/	ZT13003ZT	Switzerland	Ge et al., 2015 [44]
*L. rubrotinctus* (Peck) Redhead	JN944081	JN940295	KUNHKAS54240	China	NCBI Database
*L. sabinae* (Angelini, Justo & Vizzini) Kun L. Yang, Jia Y. Lin & Zhu L. Yang	KM983667	KM983669	ANGE306	Dominican Republic	Justo et al., 2015 [75]
*L. sardous* (Zecchin & Migl.) Migl. & Donato	KU041693	/	MCVE20105	Italy	Muñoz et al., 2015 [7]
*L. scissus* Justo, Bizzi & Angelini	NR173873	/	JBSD139953 (T)	Dominican Republic	Justo et al., 2021 [43]
*L. serenus* (Fr.) M. Asif, Saba & Vellinga	AF482871	AY176421	ecv1930L	Belgium	Vellinga et al., 2003 [48]
***L. shenyangensis** * **Z.Q. You & H.S. Yuan**	**PV460238**	**PZ235509**	**Yuan20026 (T)**	**China**	**Present study**
***L. shenyangensis***	**PV460239**	**PZ235508**	**Yuan19598**	**China**	**Present study**
*L. silvestris* (Justo, Bizzi & Angelini) M. Asif, Saba & Vellinga	NR173876	/	JBSD130934 (T)	Dominican Republic	Justo et al., 2021 [43]
*L. stillatus* (Justo, Bizzi & Angelini) M. Asif, Saba & Vellinga	NR173877	/	JBSD130937 (T)	Dominican Republic	Justo et al., 2021 [43]
*L. straminellus* (Bagl.) Narducci & Caroti	PX527025	PX527066	FJAU78063	China	NCBI Database
*L. subcrystallifer* (Z.W. Ge & Zhu L. Yang) M. Asif, Saba & Vellinga	KP205399	/	ZWGe796	China	Ge et al., 2015 [44]
*L. subhymenoderma* (Bon & A. Caball.) Kun L. Yang, Jia Y. Lin & Zhu L. Yang	KT992146	/	AC2638 (T)	Spain	NCBI Database
*L. sublittoralis* (Kühner ex Hora) Migl. & Donato	AY176442	AY176443	Vellinga2235L	Netherlands	Vellinga 2004 [5]
*L. subpurpureolilacinus* (Z.W. Ge & Zhu L. Yang) M. Asif, Saba & Vellinga	KP096233	/	ZWGe406	China	Ge et al., 2015 [44]
*L. subvolvatus* (Malençon & Bertault) M. Asif, Saba & Vellinga	KP300878	/	AMBrandsnHunga-ryL	Hungary	Ge et al., 2015 [44]
*L. sultanii* (S. Hussain, H. Ahmad & Khalid) M. Asif, Saba & Vellinga	KU647732	KU900519	FHSH115	Pakistan	Hussain et al., 2018 [46]
*L. tangerinus* (Y. Yuan & Jun F. Liang) Kun L. Yang, Jia Y. Lin & Zhu L. Yang	PQ321878	PQ319804	HTBM0679	China	Yang et al., 2024 [10]
*L. taniae* (C. Heisecke & M.A. Neves) Kun L. Yang, Jia Y. Lin & Zhu L. Yang	MT952879	/	MAN1206 (T)	Brazil	Heisecke et al., 2022 [76]
*L. tener* (P.D. Orton) M. Asif, Saba & Vellinga	GQ329043	/	MCVE751	Italy	Osmundson et al., 2013 [60]
*L. tephrolepis* Justo, Bizzi, Angelini & Vizzini	NR200350	/	JBSD130966 (T)	Dominican Republic	Justo et al., 2021 [43]
*L. thallensis* Z. Khan, Izhar & Khalid	OP972578	OQ568217	LAH37619	Pakistan	Khan et al., 2023 [77]
*L. truncatus* (Z.W. Ge & Zhu L. Yang) M. Asif, Saba & Vellinga	NR155319	/	HKAS49288	China	Ge et al., 2015 [44]
*L. turgipes* (Justo, Bizzi & Angelini) M. Asif, Saba & Vellinga	NR173880	/	JBSD130940 (T)	Dominican Republic	Justo et al., 2021 [43]
*L. umbonatus* (S. Hussain, H. Ahmad & Afshan) M. Asif, Saba & Vellinga	KU647737	KU900521	LAHSHL1	Pakistan	Hussain et al., 2018 [46]
*L. vassiljevae* (E.F. Malysheva, Svetash. & E.M. Bulakh) M. Asif, Saba & Vellinga	JX133170	/	LE289338	Russia	Malysheva et al., 2013 [67]
*L. viridariorum* (G. Muñoz, A. Caball., Salom & Vizzini) Kun L. Yang, Jia Y. Lin & Zhu L. Yang	KU041692	/	JCS296L	Spain	Muñoz et al., 2015 [7]
*L. viridiflavus* (Petch) E. Ludw.	GU574745	/	CALI10XI2004	China	Liang et al., 2010 [57]
*L. viscidulus* (Heinem.) M. Asif, Saba & Vellinga	PP756657	/	ANKAcar1305	Turkey	NCBI Database
*L. volvatus* (Bon & A. Caball.) Migl. & Donato	PV026213	/	MV161494	Italy	Migliozzi and Donato 2025 [78]
*L. wichanskyi* (Pilát) Migl. & Donato	AF482874	/	IXX1987	The Netherlands	Vellinga et al., 2003 [48]
*Rhizocybe alba* Y.X. Ding & E.J. Tian	OP626999	OP646425	KUN-HKAS123143	China	He et al., 2023 [79]
*R. vermicularis* (Fr.) Vizzini, P. Alvarado, G. Moreno &Consiglio	KJ681034	KJ681040	AH44080	Spain	Alvarado et al., 2015 [80]
*Tephrocybe ambusta* (Fr.) Donk	AF357058	/	CBS450.87	France	Hofstetter et al., 2002 [81]
*T. ambusta*	AF357057	/	CBS452.87	Switzerland	Hofstetter et al., 2002 [81]
*T. anthracophila* (Lasch) P.D. Orton	OM905952	/	BR5020155848666	Ukraine	van de Peppel et al., 2022 [20]
*T. anthracophila*	OM905953	/	K(M):164958	England	van de Peppel et al., 2022 [20]
*T. atrata* (Fr.) Donk	KP192645	/	BBF:GC98102503	France	Bellanger et al., 2015 [82]
*T. confusa* (P.D. Orton) P.D. Orton	KP192548	/	PAM00100616	France	Bellanger et al., 2015 [82]
*T. confusa*	KP192611	/	AB92-11-424	France	Bellanger et al., 2015 [82]
*T. coracina* (Fr.) M.M. Moser	OM905954	/	K(M):195497	England	van de Peppel et al., 2022 [20]
*T. coracina*	KP192632	/	BBF:GC08101102	France	Bellanger et al., 2015 [82]
*T. erosa* (Fr.) Bon	KP192541	/	PAM01101601	France	Bellanger et al., 2015 [82]
*T. erosa*	KP192634	/	GC97062013	France	Bellanger et al., 2015 [82]
*T. fibrosipes* Métrod ex Bon	KP192576	/	AB02-10-175	France	Bellanger et al., 2015 [82]
*T. fuscipes* P.D. Orton	OM905955	/	K(M):90837	England	van de Peppel et al., 2022 [20]
*T. impexa* (P. Karst.) M.M. Moser	OM905956	/	K(M):191686	England	van de Peppel et al., 2022 [20]
*T. mephitica* (Fr.) M.M. Moser	OM905958	/	K(M):196494	England	van de Peppel et al., 2022 [20]
*T. ochraceobrunnea* (Métrod ex Consiglio & Contu) P.-A. Moreau & Courtec.	KP192549	/	LIP:PAM02090608	France	Bellanger et al., 2015 [82]
*T. platypus* (Kühner) M.M. Moser	OM905961	/	BR5020153733858	Belgium	van de Peppel et al., 2022 [20]
*T. rancida* (Fr.) Donk	OM905966	OM906004	CORT012400	USA	van de Peppel et al., 2022 [20]
*T. rancida*	OM905967	OM906005	TR2017	The Netherlands	van de Peppel et al., 2022 [20]
*T. striaepilea* (Fr.) Donk	KP192647	/	BBF:GC98101106	France	Bellanger et al., 2015 [82]
*T. substriipilea* Contu & Vizzini	PX632623	/	GE22008	France	Eyssartier 2025 [83]
***T. umbonata** * **Z.Q. You & H.S. Yuan**	**PZ235487**	**PZ235512**	**Yuan21497 (T)**	**China**	**Present study**
***T. umbonata***	**PZ235488**	**PZ235513**	**Yuan21507a**	**China**	**Present study**

## Data Availability

The sequences generated in the present study were submitted to GenBank through the NCBI website (https://www.ncbi.nlm.nih.gov/; accessed on 10 March 2026), and the accession numbers were listed in Table 1. The nomenclatural information for the three new species was registered in Fungal Names (https://nmdc.cn/fungalnames/; accessed on 17 April 2026). The sequence alignments and phylogenetic trees were deposited in TreeBASE (https://www.treebase.org/; accessed on 11 May 2026). All other data supporting the findings of this study are included in the article.
